# In Vitro Suppression of T Cell Proliferation Is a Conserved Function of Primary and Immortalized Human Cancer-Associated Fibroblasts

**DOI:** 10.3390/ijms22041827

**Published:** 2021-02-12

**Authors:** Mohammed H. Abuwarwar, Alfie T. Baker, Jeffrey Harding, Natalie L. Payne, Andras Nagy, Konstantin Knoblich, Anne L. Fletcher

**Affiliations:** 1 Department of Biochemistry and Molecular Biology, Biomedicine Discovery Institute, Monash University, Clayton 3800, Australia; mohammed.abuwarwar@monash.edu (M.H.A.); alfie.baker@monash.edu (A.T.B.); 2 Lunenfeld-Tanenbaum Research Institute, Mount Sinai Hospital, Toronto, ON M5G 1X5, Canada; jharding@lunenfeld.ca (J.H.); nagy@lunenfeld.ca (A.N.); 3 Australian Regenerative Medicine Institute, Monash University, Clayton 3800, Australia; natalie.payne@monash.edu; 4 Institute of Immunology and Immunotherapy, University of Birmingham, Edgbaston B15 2TT, UK

**Keywords:** Stromal cells, T cells, cancer-associated fibroblasts, tumor microenvironment, tumor immunology

## Abstract

T cell immunotherapy is now a mainstay therapy for several blood-borne cancers as well as metastatic melanoma. Unfortunately, many epithelial tumors respond poorly to immunotherapy, and the reasons for this are not well understood. Cancer-associated fibroblasts (CAFs) are the most frequent non-neoplastic cell type in most solid tumors, and they are emerging as a key player in immunotherapy resistance. A range of immortalized CAF lines will be essential tools that will allow us to understand immune responses against cancer and develop novel strategies for cancer immunotherapy. To study the effect of CAFs on T cell proliferation, we created and characterized a number of novel immortalized human CAFs lines (Im-CAFs) from human breast, colon, and pancreatic carcinomas. Im-CAFs shared similar phenotypes, matrix remodeling and contraction capabilities, and growth and migration rates compared to the primary CAFs. Using primary isolates from breast carcinoma, colorectal carcinoma, and pancreatic ductal adenocarcinoma, we report that CAFs across major tumor types are able to potently suppress T cell proliferation in vitro. Im-CAFs retained this property. Im-CAFs are a key tool that will provide important insights into the mechanisms of CAF-mediated T cell suppression through techniques such as CRISPR-Cas9 modification, molecular screens, and pipeline drug testing.

## 1. Introduction

Immune surveillance is a monitoring process in which immune cells target transformed or malignant cells for recognition and elimination [1]. Tumors overcome immune surveillance by acquiring mechanisms to evade or suppress the immune system. T cell cancer immunotherapies have shown promising results across multiple types of malignancies, by improving the host immune response against tumors and overcoming immune evasion. Examples of these therapies are immune checkpoint blockade and chimeric antigen receptor (CAR) T cells, which respectively seek to reawaken or infuse anti-tumor T cells [2,3].

However, despite the clinical success of immune checkpoint inhibitors and CAR-T cells in treating several circulating hematologic cancers, they have not been as effective in solid tumors [4]. Reasons for this are still an active area of study; however, one key difference between these tumor types is the presence of the tumor microenvironment, also termed the tumor stroma. Emerging evidence suggests that this could be a major obstacle for immunotherapies [5].

The tumor microenvironment is comprised of cells that substantially influence tumor growth, metastasis, and therapeutic resistance, including fibroblasts, adipocytes, endothelial cells, pericytes, macrophages, lymphocytes, and other innate and adaptive immune cells as well as extracellular matrix (ECM) and basement membrane [6,7]. Immune cells such as lymphocytes and macrophages influence pro-tumorigenic and anti-tumorigenic functions through the production of an array of cytokines, chemokines, polypeptide growth factors, hormones, matrix remodeling proteases, and metabolites [8,9]. Adipocytes support tumors predominantly via the production of growth factors and cytokines, and they contribute to resistance to chemotherapies and radiotherapies [10]. Endothelial cells form new vessels via sprouting from locally pre-existing vessels to provide nutritional support and gas exchange to the growing tumor, and they have also been reported as playing important functions in resistance to radiation and chemotherapies [11,12]. Pericytes also play roles in promoting angiogenesis and contributing to resistance to antiangiogenic treatments [13].

Cancer-associated fibroblasts (CAFs) are a dominant cell type found in solid tumors, and their abundance is often associated with poor prognosis [14]. Tumors with a high proportion of CAFs associate with poor patient outcomes in breast, colorectal, pancreatic, urothelial, and oral cancers [15,16,17,18,19,20]. For example, high stroma burden in patients with colorectal carcinoma displayed the worst overall survival and relapse-free survival of any patient group [16]. Moreover, a high expression of epithelial-mesenchymal transition (EMT)/stroma-related gene led to a significantly decreased overall survival in urothelial cancer patients in comparison to low EMT patients [15]. Similarly, lung squamous cell carcinoma patients with expression of vimentin, a mesenchymal marker, have significantly reduced overall survival than patients whose tumors lack vimentin [17].

CAFs are themselves a group of heterogeneous cells that are likely mostly derived from chronically inflamed tissue-resident fibroblasts [21]. Several important roles have been ascribed to CAFs, including extracellular matrix remodeling and secretion of growth factors that regulate tumor survival, angiogenesis, tissue invasion, and metastasis [22]. Murine and human studies have shown that CAFs also play essential roles in promoting immunosuppression and evasion from immune surveillance by inhibiting effector T cell differentiation, promoting regulatory T cells (Treg) and type 2-immunity, sequestering tumor infiltrating T cells away from the tumor epithelium and reducing their migration into the TME [14,23,24,25].

CAFs are often characterized by elevated expression of markers such as alpha-Smooth Muscle Actin (αSMA), fibroblast activation protein (FAP), fibroblast-specific protein 1 (FSP1 or S100A4), Vimentin, CD90 (Thy-1), podoplanin, platelet-derived growth factor receptor-α (PDGFRα), and/or PDGFRβ, which are present in various combinations across different types of tumor and play different pro-tumorgenic functions [26,27,28]. For instance, αSMA expression is responsible for CAF contractility and formation of altered collagen structure in tumors [29]. FAP belongs to the membrane-bound serine protease family and is involved in ECM remodeling [30,31]. PDGFRs are tyrosine kinase receptors, and their expression has been shown to be associated with CAF infiltration into the tumor and tumor development [32,33]. These markers are not unique to CAFs, as they are also expressed in other cell types and in healthy tissue, and as CAFs are heterogeneous, these markers can be differentially expressed in vivo. CAFs lack expression of lineage markers for epithelium and tumor cells, e.g., epithelial cell adhesion molecule (EpCAM), endothelium and lymphatics (e.g., CD31, LYVE1), and hematopoietic cells (e.g., CD45) [18,34,35].

Primary CAFs without immortalization can only be sub-cultured for a limited number of passages. Batch differences are an important consideration as this field progresses and are likely to affect data reproducibility as well as slow down progress in the field by requiring researchers to source primary clinical tissues. Additionally, long-term culture of primary cells may impair the cell morphology, proliferation, and expression of surface markers [36,37]. Moreover, senescent cells are thought to have an altered secretory phenotype that could affect their immunosuppressive abilities [38].

In this study, we generated four novel human immortalized CAF (Im-CAFs) lines from primary human breast carcinoma, colorectal carcinoma, and pancreatic ductal adenocarcinoma tumors. We investigated their phenotypic and functional properties in comparison to their parental counterparts. The aim of this work was to create tools to dissect the mechanisms and pathways associated with CAF-driven T cell suppression, by generating CAF lines that maintain long-term cell proliferation and T cell modulating properties without compromising central CAF characteristics.

## 2. Results

**Figure 1:**
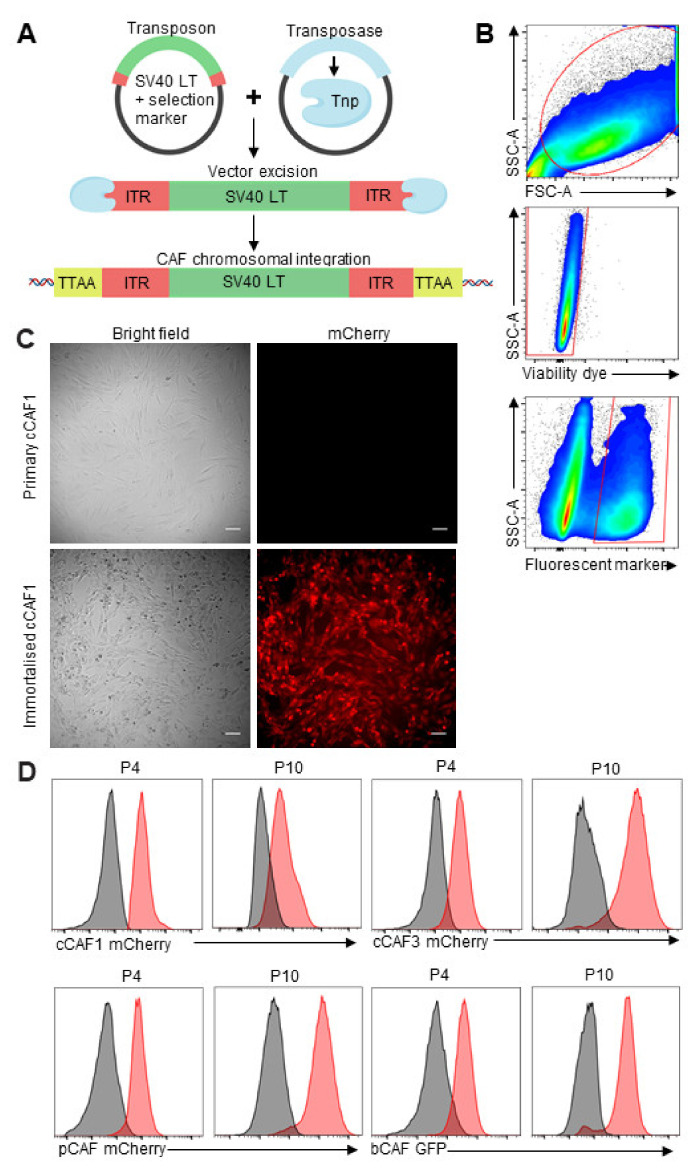
Generation of immortalized cancer associated fibroblasts (Im-CAFs). (**A**). The simian virus 40 large T (SV40LT) antigen + green fluorescent protein (GFP)/or mCherry expression plasmid was co-transfected into primary CAFs with the transposase expression plasmid. Once inside the cell, the transposase (Tnp, blue) is expressed and recognizes the inverted terminal repeat (ITR, red) sequences flanking the SV40LT and fluorescent marker sequences. The transposase excises the gene of interest, fluorescent marker and flanking ITR sequences from the expression plasmid and integrates it randomly into TTAA recognition sites throughout the genome (yellow). (**B**). Transfected CAFs were sorted by fluorescent selection marker using fluorescence activated cell sorting (FACS). bCAF sample shown; representative of 4 cell lines. (**C**). Brightfield and immunofluorescence of primary and immortalized cCAF1 cell lines. Scale bar is 100 μm. (**D**). Expression of mCherry and GFP in immortalized CAFs at different passages. P = passage number. Red = transfected, grey = untransfected.

We obtained primary human CAFs from breast carcinoma (bCAFs), colorectal carcinoma (cCAFs), and pancreatic adenocarcinoma (pCAFs) (patient information shown in Supplementary Table S1). To generate immortalized CAFs, we introduced the simian virus 40 large T (SV40LT) antigen and a fluorescent selection marker (either mCherry or green fluorescent protein (GFP)) via the piggyBac transposon system [39,40]. The gene encoding SV40LT was first cloned into pDONR221 and then subcloned into a piggyBac expression plasmid upstream of an internal ribosome entry site (IRES)-GFP or -mCherry site (Figure 1A). Transfected CAFs were then sorted by fluorescent marker expression using Fluorescence Activated Cell Sorting (FACS) (Figure 1B). Immortalized breast CAFs expressing GFP were termed Im-bCAF; immortalized pancreatic and colorectal CAF expressing mCherry were termed Im-pCAF, Im-cCAF1, and Im-cCAF3 (established from separate cCAF donors), respectively, to differentiate them from parental cell isolates. The immortalized lines showed stable expression of the fluorescent markers over time, outlasting parental primary cells with stable growth out to passage 10 (Figure 1C,D).

**Figure 2:**
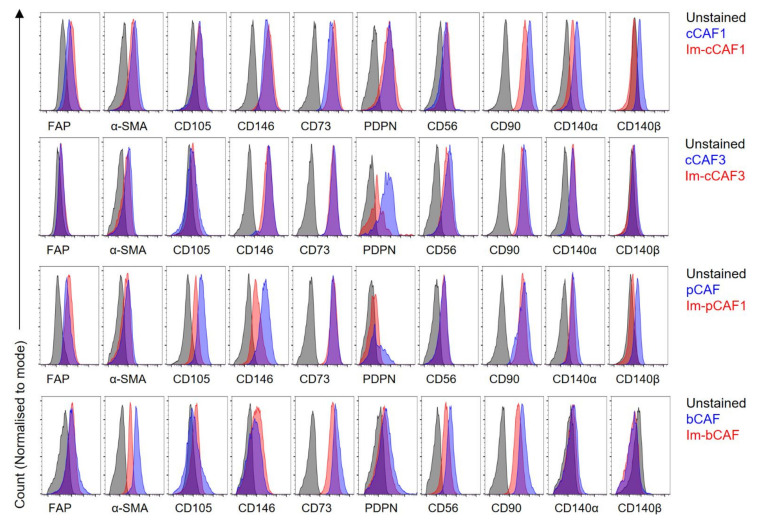
Phenotype of immortalized CAFs. Primary CAFs at passage 3 and immortalized (Im-)CAFs at passage 4–5 were phenotyped for a range of characteristic CAF markers. Histograms shown are gated on non-debris, viable cells, and mCherry+ for Im-cCAF1, Im-cCAF3, and Im-pCAF, or GFP+ for Im-bCAF, with no fluorescent selection for primary cells. Data represent 2–3 independent experiments.

Next, we examined the phenotype of immortalized cell lines compared to parental primary CAFs to ensure that expression of major markers was retained. A range of characteristic surface and intracellular markers were used to phenotype the primary and immortalized CAFs including Podoplanin (PDPN), fibroblast activation protein (FAP), CD105 (Endoglin), CD146 (Melanoma cell adhesion molecule (MCAM), Ecto-5′-nucleotidase (CD73), α-smooth muscle actin (αSMA), CD56 (Neural-cell adhesion molecule, NCAM), Platelet-derived growth factor receptor- α and β (CD140α and CD140β), and CD90 (Thy1). Immortalized cells maintained a similar surface phenotype of characteristic CAF markers compared to parental lines (Figure 2), including past the point of primary cell senescence (bCAF1, Figure 2; cCAF1, Supplementary Figure S1). The study was not constructed to robustly compare surface expression levels; however, we did observe a conserved reduction in both CD140a and CD140b expression in the line cCAF1, including at later passages (Supplementary Figure S1). In this line, it is likely to denote an overall reduction in expression of the platelet derived growth factor receptor, which is made up of the CD140a and b subunits (homo- or hetero-dimeric), but it was not observed for other lines. Immortalized cells broadly maintained a CAF phenotype. 

**Figure 3:**
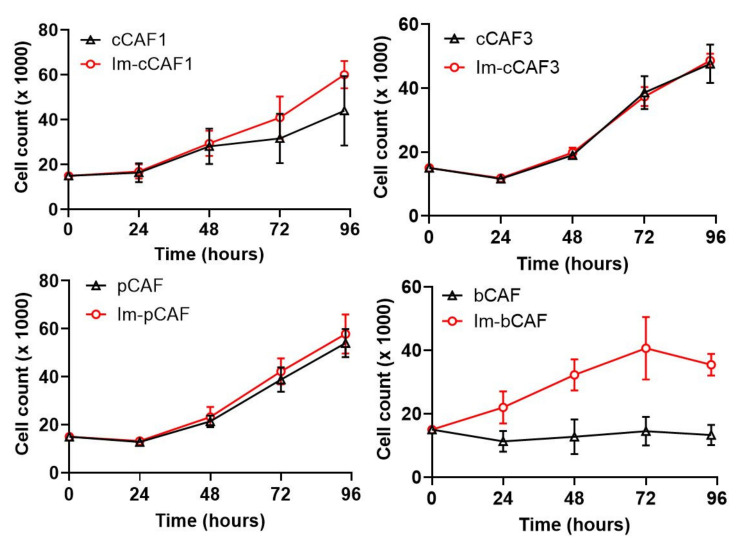
Growth assay of primary CAFs and immortalized (Im-)CAFs. 15.0 × 10^3^ cells of the indicated Im-CAF (passage 4) or primary (passage 3) cell lines were plated and then harvested at the indicated time points. The line graph depicts the mean ± SD of 6 replicates from 2 independent experiments.

Moving forward with an assessment of CAF function, a potential concern with immortalization using SV40 LT is the possibility of genomic changes, which may alter the growth rate of the cell lines; therefore, we next compared the growth rate of the immortalized lines versus the primary CAFs. We found the growth rate was similar between Im-cCAF1, Im-cCAF3, and Im-pCAF lines to compare expression with their parental CAFs. The doubling time for primary bCAFs, which appeared to be reaching senescence, was strongly outpaced by Im-bCAF (Figure 3). In this case, the rapid growth rate of the immortalized sample permitted work with these cells that was not possible with the primary isolate.

**Figure 4:**
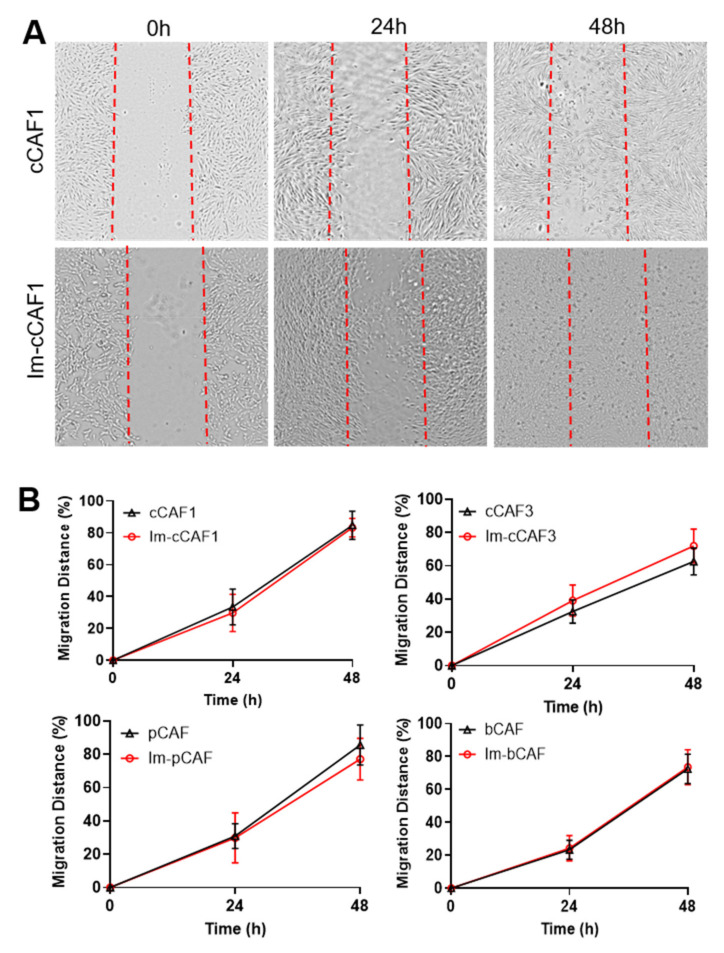
Immortalized CAFs are migratory. (**A**) Representative images of migration of CAFs across a scratch at 0, 24, and 48 h after the scratch was made. Dotted lines depict where the scratch was made. (**B**) The proportion of the original scratch area closed after 24 and 48 hrs. Data depicts evaluations of primary cells at P3 and immortalized cells from P4–P9. The line graph represents the mean value ±SD of 10–16 replicates per cell line, from 2 independent experiments.

In order to show that the immortalization process did not result in functional impairment in motility, we investigated the migration rate between the immortalized CAFs and primary CAFs; wound healing/scratch assays were performed [41]. Primary and Im-CAFs were seeded in 24-well plates until they reached confluency, then they were serum-starved for one hour to ensure cell cycle synchronization before a scratch was performed. Scratches were monitored for 48 h, which demonstrated that immortalized CAFs closed the scratch at a similar migration rate to primary CAFs (Figure 4).

**Figure 5:**
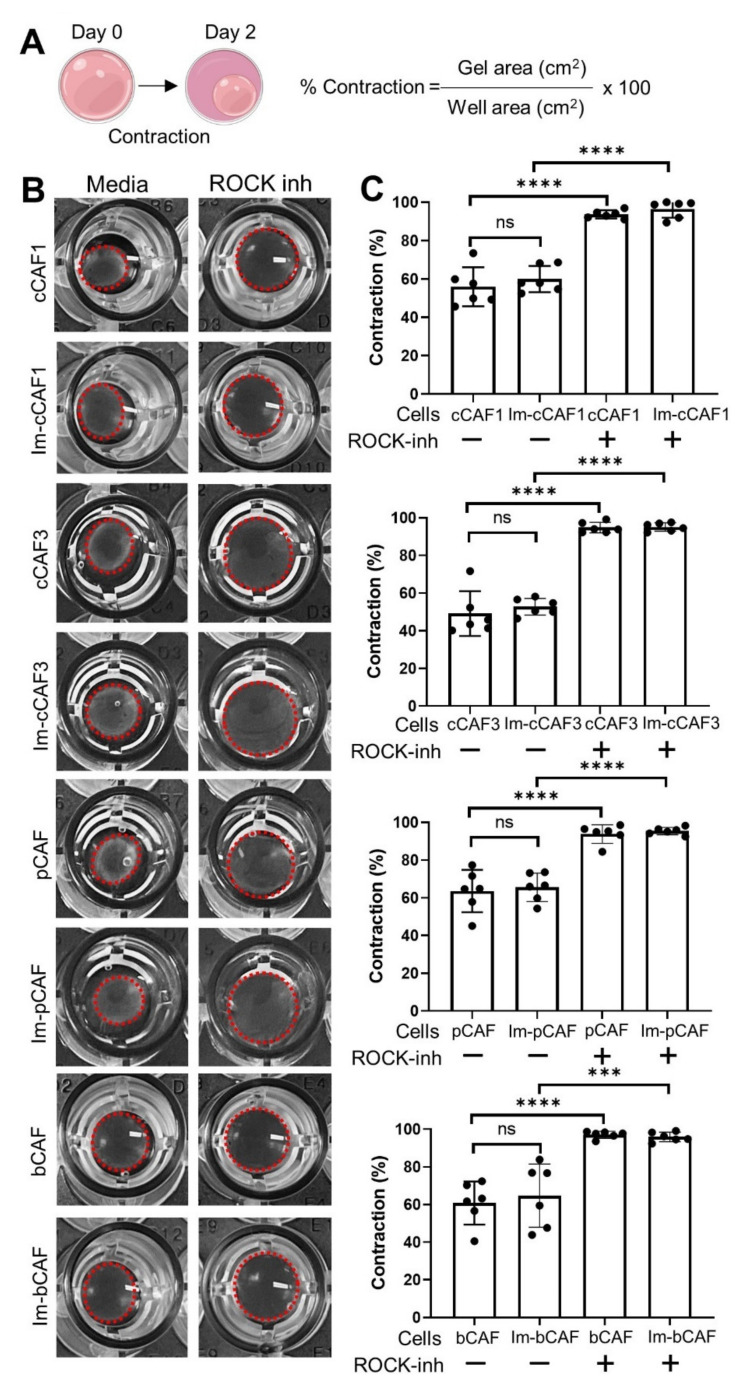
Immortalized CAFs are contractile. Primary CAFs and immortalized CAFs were seeded into a collagen gel with or without Rho-associated protein kinase (ROCK) inhibitor (100 mM) for 48 h. (**A**). Schematic representation of gel remodeling. Gels were detached, the gel contraction was digitally photo-documented, and % contraction calculated as shown. (**B**). Contraction was measured as a % reduction of gel surface after 48 h. (**C**). Pooled data representing mean values ± SD of 6 replicates from 2 independent experiments. Primary cells were evaluated at passage 3; immortalized cells at passage 4–5. One-way ANOVA, ns: non-significant, *** *p* < 0.001, **** *p* < 0.0001.

Mechanical remodeling of the ECM by CAFs is a crucial contributor to tumor cell migration and invasion [42]. To assess whether immortalized CAFs are capable of matrix remodeling, we performed collagen gel contraction assays. Primary and immortalized CAFs were seeded in type I collagen gels, and contraction was observed using cells treated with Rho-associated, coiled-coil containing protein kinase (ROCK) inhibitor as a negative control for contraction (Figure 5). Contraction of the gels was then monitored over 48 h, which revealed that Im-bCAF, Im-cCAF1, Im-cCAF3, and Im-pCAF were able contract the gels to an equal extent as primary cells. 

**Figure 6:**
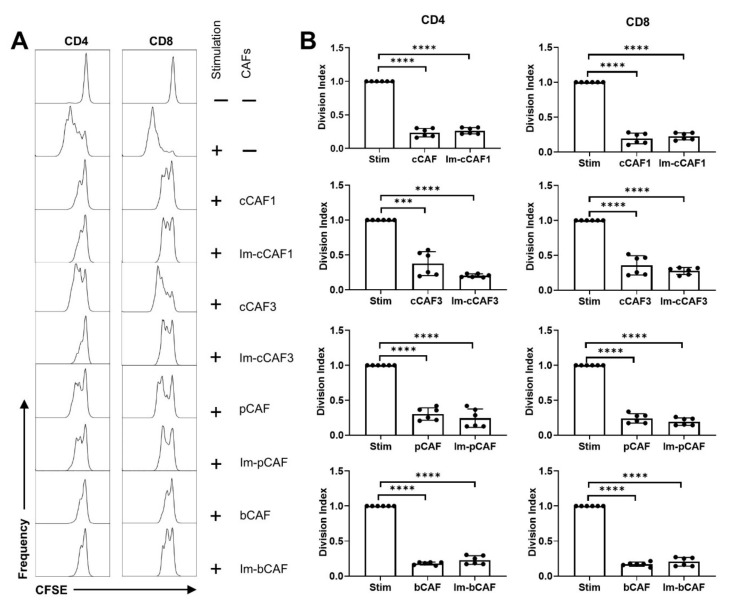
Human primary CAFs and immortalized CAFs inhibit T-cell proliferation. (**A**). Carboxyfluorescein succinimidyl ester (CFSE)-labelled peripheral blood mononuclear cells (PBMCs) (5 × 10^5^) were cultured with or without CAFs grown to 90% confluency in 96-well plates, with or without anti-CD3/CD28/CD2-coated activation beads. After 96 h, cells were harvested and analyzed using flow cytometry. Histograms depict T-cell CFSE staining on plots gated for CD3 and CD4 or CD8. (**B**). Normalized division index of gated CFSE+ T cells, which were cultured for 96 h unstimulated or stimulated with anti-CD3/CD28/CD2-coated beads, with or without primary or Im-CAFs. Data are normalized to stimulated T cell controls and represent mean values ± SD of 6 replicates from 2 independent experiments. Data represent primary cells evaluated at passage 3 and immortalized cells at passages 4–13. Statistics: one sample *t* test, *** *p* < 0.001, **** *p* < 0.0001).

Immortalized lines were then compared with primary cells for the ability to suppress T cell activation and proliferation, which is an immunomodulatory function attributed to primary CAFs [43]. This was tested using Carboxyfluorescein succinimidyl ester (CFSE)-labelled T-cells from healthy donors incubated with or without human primary or immortalized CAFs for 96 h, followed by flow cytometric analysis to determine the proliferation index of T cells. Results showed that the immortalized CAFs were able to potently suppress T cell proliferation at a level comparable with primary parental cells (Figure 6). This was conserved past senescence for all cell lines, and out to passage 13 for at least one line (Supplementary Figure S2). Immortalized CAFs are therefore likely to represent a useful tool to dissect the mechanisms and pathways associated with CAF-driven T cell suppression.

## 3. Discussion

Within a tumor, fibroblasts and myofibroblasts are commonly referred to as cancer-associated fibroblasts (CAFs), which are one of the most abundant cell types found in the tumor stroma [14]. CAFs simultaneously construct and remodel the ECM by expressing collagens and other fibrous proteins, alongside proteolytic enzymes, such as matrix metalloproteases, which degrade the ECM [22]. CAFs have crucial functions in recruiting and regulating leukocyte infiltration and inflammation through production of growth factors, cytokines, and chemokines [14,44].

CAFs create an immunosuppressive TME that indirectly promotes cancer growth [44] and may directly contribute to immunotherapy resistance [27]. CAFs can modulate both innate and adaptive immune systems by direct recruitment of immune cells via release of chemokines and cytokines including CCL2 [4]. Moreover, ECM remodeling by CAFs may impede trafficking of T cells leading to more restrained tumor immunity [45]. CAFs also suppress the function of T cells and inhibit their tumor cell killing capacity by limiting T cell infiltration into the tumor and inhibition of the T cells cytotoxic activity within the TME [46,47,48,49].

In our hands, primary human CAFs were sub-cultured for 3–8 passages before they stopped proliferating, reaching replicative senescence. CAFs undergoing senescence may undergo transcriptional and functional changes that may affect the phenotypes under study. Thus, for experiments that require long-term culture, such as genome editing, immortalization is imperative. In the present study, we successfully immortalized CAFs from different patients by introducing SV40LT via the efficient virus-free piggyBac transposon system [50,51]. To date, several immortalized genes have been used to immortalize cell lines, amongst which SV40LT is one of the most investigated genes. The SV40LT inhibits tumor suppressor p53 and retinoblastoma suppressor gene (RB) to prolong the cell cycle and promote cell immortalization [52,53].

Phenotypic changes are possible after immortalization with the SV40LT gene. The Im-CAFs we created expressed all of the CAF markers used in this study, indicating that the immortalization process with SV40LT did not affect their major phenotype, as evaluated by flow cytometry.

Another key function of CAFs is ECM remodeling through contractility of CAF [42,54]. This is a hallmark of the myofibroblast phenotype [22,55], which can be assessed via collagen gel contraction capability in vitro [56,57]. Our results clearly demonstrate that Im-CAFs are capable of remodeling ECM to contract a collagen gel, again confirming that Im-CAFs shared key phenotypes with the primary CAFs. Im-CAFs also showed a similar migratory capability compared to the parental CAFs. To investigate the growth rate of the immortalized lines, CAFs and Im-CAFs were cultured and monitored for 4 days. Im-CAFs expanded as fast as the primary CAFs, while the primary bCAFs grew slower than Im-bCAFs and had likely reached senescence, highlighting the value of immortalization in permitting continued study beyond the lifespan of the parental primary cell line. Importantly, immortalized cell growth, migration, and T cell suppression were maintained at late passages 9–13, representing 10,000-fold expansion after primary cells senesced.

Data shown here and from others illustrates that CAFs derived from breast, pancreatic, and colorectal tumors inhibit proliferation of human T lymphocytes in vitro [43,58,59]. Our results show that both Im-CAFs and their parental CAFs are capable of inhibiting T cell proliferation, and for the first time that CAFs exerted a conserved T cell suppressive effect across various solid tumor types, at least in vitro. However, T cell suppression is not unique to CAFs; rather, it appears to be a conserved property of activated fibroblasts [60,61,62,63,64], supporting the possibility that this observation is likely to be relevant to human biology and the development of new therapeutics. These early proof-of-concept findings indicate that the Im-CAFs are a key tool that can provide further insights into the underlying mechanisms of CAF-mediated T cell suppression, and to explore further effects on T cell function, including changes to methylation state, differentiation, cytokine production, and tumor cell killing. Further exploration of immortalized CAF phenotypes including transcriptomics and secretory profiles would also be insightful.

Identifying molecules that affect T cell activation, differentiation, and function in cancer is extremely important to the development of effective immunotherapies. A substantial body of literature has shown that a high number of tumor-infiltrating lymphocytes correlates with an increased overall survival in several cancers [65,66,67,68]. Additionally, a durable clinical response to CAR-T therapy correlates with extensive proliferation of CAR-T cells [69]. Moreover, the presence of CAFs is known to create an immunosuppressive TME that suppresses the function and proliferation of T cells and skews T helper differentiation towards Th2 and Treg subtypes [25,27,70,71,72,73]. Our finding that the deregulation of T cell proliferation in the presence of CAFs is a conserved function of CAFs from a variety of tumor types may be highly relevant to cancer immunotherapy. Preclinical data has reported that several growth factors and cytokines released by CAFs are involved in poor T cell outcomes, including prostaglandin E2, indoleamine-2,3-dioxygenase, and transforming growth factor beta [23,74,75,76]. The use of immortalized CAFs will further our understanding of these mechanisms involved in CAF-mediated T cell regulation, including effector function, T helper differentiation, and T cell proliferation, through techniques such as CRISPR-Cas9 modification, molecular screens, and drug testing.

This early proof-of-concept study shows that our immortalized CAFs from multiple tumor types share important characteristics with their parental cells, including expression of CAF markers, migration and contractility rates, and the suppression of T cell proliferation. These properties indicate that the immortalized CAFs could overcome several of the limitations of primary cells by providing a reproducible and robust model. These immortalized CAFs represent tools for future studies exploring genes controlling CAF-T cell interactions, using genetic engineering technologies. Our data further show that the suppression of T cell proliferation is a conserved function of CAFs from multiple tumor types including breast, colorectal, and pancreatic cancers.

## 4. Materials and Methods

### 4.1. Primary Cells

Primary human cells from breast (bCAFs), colorectal (cCAFs), and pancreatic CAFs (pCAFs) were purchased from Asterand Bioscience (now BioIVT) and used to generate immortalized cells. All cells were maintained in Alpha Modified Eagle Medium (αMEM) (Sigma-Aldrich) with 10% fetal bovine serum (FBS). Blood from healthy donors was obtained from LifeBlood Australia under material supply deed 18-06VIC-10. All work was conducted with the approval of Monash Human Research Ethics Committee, where CAFs were immortalized under project number 18,025, and blood was obtained and used under project number 11,939. All work was conducted in accordance with institutional guidelines and according to the principles expressed in the Declaration of Helsinki.

### 4.2. Construction of PiggyBac Transposon Vectors

A cassette containing a cytomegalovirus early enhancer/chicken β-actin (CAG) promoter and downstream gateway recombination site (Addgene #20960) was ligated into a backbone containing a 5′ and 3′ piggybac long-terminal repeats and an SV40 PA terminator (Addgene #80758) at SgfI/EcoRV. Then, using extension PCR, loxP-flanked IRES-GFP and IRES-mCherry fragments were generated and ligated into the piggyBac-CAG-Gateway vector at Bsu361/SphI. Finally, SV40LT was cloned into these plasmids by gateway recombination.

### 4.3. Establishment of Immortalized CAFs

Early passage primary CAFs (<3 passages) were seeded at a density of 5 × 10^4^ cells/well in 6-well plates in complete media (αMEM/10% FBS). Using Lipofectamine 3000, CAFs were co-transfected with a piggyBac expression vector, which expresses SV40LT with GFP or mCherry, and the piggyBac transposase expression vector, hyPBase (The Sanger Center, pCMV-hyPBase). Four days after transfection, and after refreshing media, the cells were cultured until confluency. Transfected CAFs were then cell-sorted by GFP or mCherry expression using FACSAria machines and FACSDiva software (all from BD Bioscience, San Jose, CA, USA) and further expanded in complete media, testing for maintained fluorescent marker expression at regular intervals.

### 4.4. Scratch Assay

The migratory behavior of CAFs was measured using a common in vitro wound healing assay [77]. CAFs were seeded at a density of 5 × 10^4^ cells/well in 24-well plates and cultured until confluent. Media was replaced with serum-free αMEM for 2 h to synchronize cell cycling. A scratch was made through the cell monolayer with a 1000 µL pipette tip. The wounded monolayers were gently washed twice with the phosphate-buffered saline (PBS) 1× to remove the debris or the floating cells. The cells were then cultured in 1 mL/well complete media. Scratches and cells were imaged at 0, 24, and 48 h with a Leica DMi8 microscope, and the images were analyzed via ImageJ software, using the “MRI Wound Healing Tool” [78]. Migration distance was measured using the following equation: (area of gap (cm^2^) at 24 or 48 h)/(area of gap (cm^2^) at 0 h).

### 4.5. T Cell Suppression Assay

CAFs (2 × 10^4^) were plated in a 96-well flat-bottom plate in complete media and allowed to adhere for 4 h. Human peripheral blood mononuclear cells (PBMCs) from healthy donors were isolated from whole blood using a density gradient Lymphoprep (Stem Cell Technologies, Canada), according to the manufacturer’s instructions, and then counted using a hemocytometer and Trypan Blue viability dye. PBMCs were then labelled with 5 μM CFSE, as previously described [79]. PBMCs (5 × 10^5^) were co-cultured with CAFs and T cell stimulating beads conjugated to anti-CD2, anti-CD3, and anti-CD28 antibodies (1 bead/4 T cells) (Miltenyi Biotec, NSW, Australia). The final volume per well was 200 μL, and cells were incubated for 96 h in complete media with 1% penicillin-streptomycin (Sigma-Aldrich, NSW, Australia).

### 4.6. Collagen Gel Contraction Assay

CAFs at a final concentration of 1 × 10^4^ cells/mL were cultured in collagen lattice constructed from 330 μL of a neutralized rat tail Collagen I (final concentration 1 mg/mL and 33% *v*/*v*) (Merck Millipore, Victoria, Australia), 33 μL of 10× αMEM stock (i.e., a 1:10 dilution with the volume of collagen added), made in house from αMEM powder (Invitrogen), and 637 μL complete media containing cells to be plated, for a final volume of 1ml. Gel was mixed on ice, and 100 μL was plated in 96-well plates. The resultant mixture was incubated for 2 h at 37 °C to induce gelation, followed by addition of 100 μL of complete media per well with or without 100 μM ROCK inhibitor (Y-27632, Stem Cell). Gels were dislodged from the walls of the well and photographed after 72 h of incubation. Contraction was measured using the following equation: area contracted gel (cm^2^)/area well (cm^2^) * 100 = % contraction.

### 4.7. Cell Proliferation

CAFs were seeded into 12-well plates at a density of 15 × 10^4^ cells per well and supplemented in complete media with 1% penicillin-streptomycin. Cell proliferation was assessed at 24, 48, 72, and 96 h by counting the number of cells after trypan blue staining (Invitrogen, Victoria, Australia).

### 4.8. Flow Cytometry

Cells obtained from suppression or phenotyping assays were stained at a density of 0.5–5 × 10^5^ cells per sample in 50 μL volume of FACS buffer (PBS containing 0.5% Bovine Serum Albumin (BSA), 2mM EDTA, and 0.1% sodium azide). Cells were stained with 30 μL of Zombie Aqua viability dye (Biolegend, San Diego, CA, USA); prepared at a dilution of 1:1000 in PBS) for 10 min in the dark. Cells were washed using 150 μL of FACS buffer, centrifuged, and decanted. For surface marker staining, the pellets were resuspended in 30 μL of appropriate antibody cocktail in FACS buffer and incubated for 15 min on ice in the dark to stain for surface markers, followed by a washing step. Cells were fixed with 100 μL of fixative (BD Cytofix, BD Biosciences, San Jose, CA, USA) and incubated for 15 min on ice in the dark, and either washed with 100 μL of FACS buffer or, for intracellular staining, permeabilization buffer (prepared at 1/10 with Milli Q water; BD Cytoperm), followed by 30 μL of antibody in permeabilization buffer for 30 min on ice. Cells were washed, resuspended in FACS buffer, and filtered through 70 μm nylon mesh. Samples were acquired on a BD Fortessa X20 (BD Biosciences, San Jose, CA, USA) and analyzed using FlowJo (v10) (BD Biosciences, San Jose, CA, USA). The CAF antibody panel included mouse anti-human CD146 (BV650, P1H12), mouse anti-human CD73 (BV785, AD2), rat anti-human PDPN (Alexa Fluor 647, NC-08), mouse anti-human CD105/Endoglin (PerCP/Cy5.5, 43A3), mouse anti-human PDGFRα (PE/Cy7, 16A1), mouse anti-human PDGFRβ (PE, 18A2), mouse anti-human CD56/NCAM (APC/Cy7, 5.1H11), mouse anti-human CD90/Thy1 (PE/Cy5, 5E10), mouse anti-human CD3 (Pacific Blue, HIB19), rat anti-human CD4 (Alexa Fluor 647, NC-08), and mouse anti-human CD8α (Biotinylated, UCHT1), which were all obtained from Biolegend, San Diego, CA, USA. Mouse anti-human FAP (Alexa Fluor 488, 427819) and mouse anti-human α-SMA (Alexa Fluor 700, 1A4) were both obtained from R&D Systems, Minneapolis, MN, USA.

### 4.9. Statistical Analysi

Data are presented as mean ± standard deviation. Data analysis was performed using Graph Stat Prizm software. For all analyses, a *p*-value of <0.05 was considered statistically significant. One-way ANOVA was used to compare three or more groups of normally distributed data, and a one sample *t*-test was used where data were normalized to a comparator group.

## Data Availability

The data presented in this study are available on request from the corresponding authors.

## Supplementary Materials

The following are available online at https://www.mdpi.com/1422-0067/22/4/1827/s1, Figure S1: Phenotype of immortalized cCAF1 at early and late passages, Figure S2: Immortalized cCAF1 maintain suppression of T-cell proliferation at late passages, Table S1: Patient Information.

## Abbreviations

CAF	Cancer-associated fibroblast
Im-CAFs	Immortalized CAFs
CAR	Chimeric antigen receptor
bCAFs	Breast carcinoma CAFs
cCAFs	Colorectal carcinoma CAFs
pCAFs	Pancreatic adenocarcinoma CAFs
Im-bCAFs	Immortalized breast carcinoma CAFs
Im-cCAFs	Immortalized colorectal carcinoma CAFs
Im-pCAFs	Immortalized pancreatic adenocarcinoma CAFs
SV40LT	Simian virus 40 large T
IRES	Internal ribosome entry site
ITR	Inverted terminal repeat
FACS	Fluorescence Activated Cell Sorting
PDPN	Podoplanin
FAP	Fibroblast activation protein
αSMA	α-smooth muscle actin
CD146/MCAM	Melanoma cell adhesion molecule
CD105	Endoglin
CD73	Ecto-5′-nucleotidase
CD56/NCAM	Neural-cell adhesion molecule
CD140α	Platelet-derived growth factor receptor- α
CD140β	Platelet-derived growth factor receptor- β
CD90/Thy1	Thymus cell antigen 1
ROCK	Rho-associated, coiled-coil containing protein kinase
CFSE	Carboxyfluorescein succinimidyl ester
RB	retinoblastoma suppressor gene
