# Supplementary material for: In Vitro Suppression of T Cell Proliferation Is a Conserved Function of Primary and Immortalized Human Cancer-Associated Fibroblasts
Source: Int J Mol Sci. 2021 Feb 12;22(4):1827. doi: 10.3390/ijms22041827 (PMC7918788; doi:10.3390/ijms22041827)
Supplement: Supplementary file 1 [file ijms-22-01827-s001.zip › ijms-1008689-supplementary material/ijms-1008689-Figure S1S2.docx]

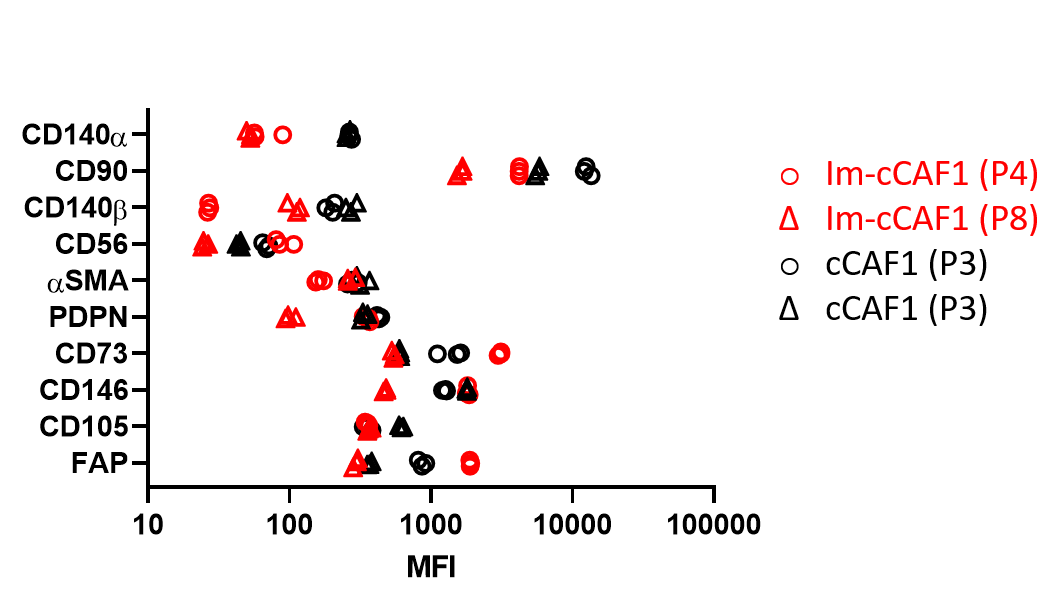


**Figure S1.** **Phenotype of immortalized cCAF1 at early and late passages.** Primary at passage 3 and Im-CAFs at passage 4 or 8 were phenotyped for a range of characteristic CAF markers. Pooled data representing MFI values of six replicates from two independent experiments.


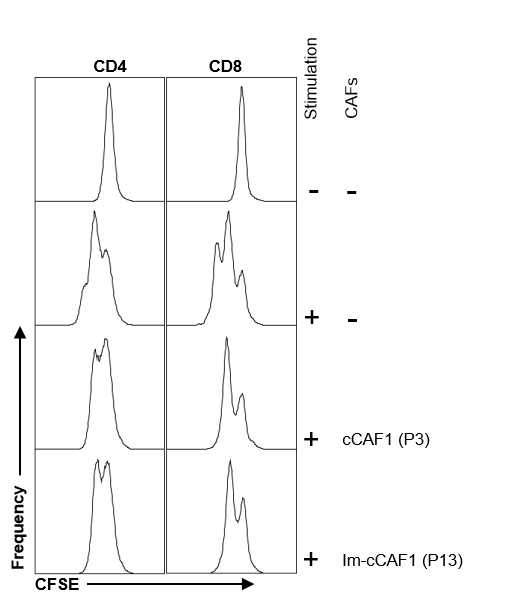


**Figure S2. Immortalized CAFs maintain suppression of T-cell proliferation at late passages**. (**a**). CFSE-labelled PBMCs (5 × 10^5^) were cultured with or without primary colorectal (c)CAFs at passage 3, or immortalized cCAFs at passage 13. CAFs were grown to 90% confluency in 96-well plates, with or without anti-CD3/CD28/CD2-coated activation beads. After 96 h, cells were harvested and analyzed using flow cytometry. Histograms depict T-cell CFSE staining on plots gated for CD3 and CD4 or CD8.
