# Supplementary material for: In Vitro Suppression of T Cell Proliferation Is a Conserved Function of Primary and Immortalized Human Cancer-Associated Fibroblasts
Source: Int J Mol Sci. 2021 Feb 12;22(4):1827. doi: 10.3390/ijms22041827 (PMC7918788; doi:10.3390/ijms22041827)
Supplement: Supplementary file 1 [file ijms-22-01827-s001.zip › ijms-1008689-supplementary material/ijms-1008689-Table S1.pdf]

Supplementary Table 1

Cell line	Tissue	Clinical Diagnosis (Specimen)	Genetic analysis	AJCC/UICC Stage	AJCC/UICC Stage Group	Age At Excision	Sex	Ethnicity	BMI	Medications	Clinical Test Results
bCAF	Breast	Infiltrating lobular carcinoma of the breast	PIK3CA:Neg, Her-2/neu: Negative, ER: Positive(40%), PR: Negative(<1%)	T3N3MX	IIIC	73	Female	Caucasian	36.68	Aripiprazole (2 mg,1/day,Current Use), Ascorbic acid (500 mg,2/day,Current Use), Buspirone (10 mg,1/day,Current Use), Citalopram (40 mg,1/day,Current Use), Multivitamin (1/day,Current Use), Omeprazole (20 mg,1/day,Current Use), Trazodone (25 mg,1/day,Current Use)	Her-2/neu: Negative, ER: Positive(40%), PR: Negative(<1%)
cCAF1	Sigmoid colon	Adenocarcinoma, mucinous type of the large intestine		T4aN0MX	IVA	60	Male		26.26	Humulin (2-5 u,1 every 6 hours,Current Use), Pantoprazole (45 mg,2/day,Current Use), Ciprofloxacin (400 mg,1/day,Current Use), Metronidazole (500 mg,1 every 8 hours,Current Use), Magnesium hydroxide and aluminum hydroxide (30ml ud,Every 4 hours,Current Use), Melatonin (3 mg,Before bed,Current Use), Hydromorphone (0.5 mg,Every hour, as needed,Current Use), Ondansetron (4 mg,Every 8 hours as needed,Current Use), Enoxaparin (4 mg,Every 24 hours,Current Use)	Fasting Plasma Glucose: 150(mg/dl)
cCAF3	Cecum	Adenocarcinoma of the large intestine		T3N2aM0	IIIB	80	Male	Caucasian	27.78	Allopurinol (300 mg,1/day in the morning,Current Use), Aspirin (325 mg,1/day as needed,Current Use), Fenofibrate (160 mg,1/day in am,Current Use), Multivitamin with minerals (1 tab,1/day,Current Use), Simvastatin (40 mg,Before bed,Current Use)	CEA: 16.8(ng/dl)
pCAF	Pancreas	Pancreatic ductal adenocarcinoma		T3N1MX	IIB	81	Female	Caucasian	26.56	Tramadol (50 mg,as needed,Current Use), Hydroxyurea (500 mg ,2/day,Current Use), Losartan (50 mg,1/day,Current Use), Atorvastatin (40 mg,1/day,Current Use), Aspirin (325 mg,1/day,Current Use), Motrin (800 mg,as needed,Current Use), Cyclobenzaprine (5 mg,as needed,Current Use)	Glucose: 142(mg/dL), Fasting Plasma Glucose: 145(mg/dL)
